# The challenge of healthcare big data to China’s commercial health insurance industry: evaluation and recommendations

**DOI:** 10.1186/s12913-022-08574-2

**Published:** 2022-09-22

**Authors:** Jun Wu, Jiajun Qiao, Stephen Nicholas, Yunqiao Liu, Elizabeth Maitland

**Affiliations:** 1 Dong Fureng Institute of Economic and Social Development, Wuhan University, Wuhan, China; 2 National Institute of Management and Commerce, Eveleigh, Australia; 3 University of Newcastle, Callaghan, Australia; 4 University of Liverpool, Liverpool, UK

**Keywords:** Commercial health insurance, Healthcare big data, PBM, DRGs

## Abstract

**Background:**

China’s social medical insurance system faces challenges in financing, product coverage, patient health responsibility sharing and data security, which commercial health insurance companies can help address. Confronting accelerated population aging, the rapid increase of patients with chronic diseases and the maternal and child healthcare needs created by the three-child policy, the Chinese government has encouraged the development of commercial health insurance. But China's commercial health insurance companies face financial sustainability problems, limited product ranges and high operating costs. At the same time, the informatization level of China's healthcare industry, and the value of healthcare big data, is increasing. We analyze and describe the potential application of healthcare big data in the life cycle of China's commercial health insurance system and provide specific action plans for Chinese commercial health insurance companies; identify the challenges to commercial health insurers; and make recommendations for the application of big health data by commercial health insurers. Our recommendations inform healthcare policy makers on the development of commercial health insurance and the improvement of the healthcare financing system. We not only verify the value of healthcare big data, but also identify specific ways that healthcare big data plays in the development of commercial health insurance. Based on the research results, we recommend new policies for government and new uses of healthcare big data for commercial health insurance institutions. The benign development of commercial health insurance will improve the level of health services in China.

**Methods:**

By interviewing health insurance managers (including actuaries, product managers, business executives, information technology medical workers, and commercial health insurance personnel) and by accessing research papers, industry reports, news reports and public information disclosure documents about commercial health insurance, we describe the impact of healthcare big data on the life cycle of commercial health insurance products and processes.

**Results:**

We identify the issues and challenges of commercial health insurers in the use of healthcare big data, and advance specific strategies to expand the use of healthcare big data. In the life cycle of commercial health insurance products, healthcare big data can improve premium income, control medical costs and increase operational efficiency. First, healthcare big data can increase premiums, products and services by attenuating moral hazard and adverse selection problems, where high quality clients over-pay and high-risk clients underpay for health insurance. Second, healthcare big data can reduce medical expenses compensation pay-outs by promoting the establishment of a management medical system. Finally, the use of healthcare big data improves operational efficiency by increasing payment speeds, identifying fraud and increasing claim verification processes through automating payments and reducing offline processes. We discuss the obstacles to obtain healthcare big data confronting commercial health insurance companies. The sharing and data mining of healthcare big data brings privacy risks to the insured and there are significant differences in data standards and quality of healthcare big data that limit the application of healthcare big data in commercial health insurance. We recommend that national, regional and local government departments coordinate policies to facilitate the cooperation between commercial health insurance companies and regional healthcare big data platforms. In terms of technology, we recommend the establishment of data sharing platforms and data exchange mechanism across institutions and regions according to nation-wide standards and specifications. Government management departments should establish healthcare big data standards and specification system, promote the construction of healthcare big data and ensure the integrity, authenticity and reliability of health data. We recommend data quality continuous improvement and management mechanisms that combine technology and management. Government regulation should oversee commercial health insurance institutions and establish data security management systems to monitor and supervise the privacy of personal data.

**Conclusions:**

Healthcare big data can play an important role in the development of China's commercial health insurance industry. Healthcare big data can increase commercial health insurers’ financial viability while providing improved, and cost-effective, products and services. By providing more and better information to insurers, healthcare big data attenuates the asymmetric information problem, addressing moral hazard and adverse selection problems. By combining hospital and medical organization management information systems with insurers’ data management, healthcare big data can help insurers set sustainable premiums, control medical costs and promote operational efficiency. At present, the informatization degree of China's healthcare industry remains limited. To improve the performances, products and services of commercial health insurers, we recommend government reforms in healthcare big data, such as expanding medical industry cooperation; further developing the processes of applying healthcare big data; augmenting data sharing; addressing privacy risks; setting data standards; and improving data quality.

## Introduction

Since 1978, the Chinese government has been reforming the health system, including social medical insurance. Covering more than 1.35 billion people, and 95% of the population [1], China’s 2009 health insurance system was defined by three basic social medical insurance programs: the urban employee basic medical insurance (UEBMI) covering employed urban residents; the urban resident basic medical insurance (URBMI) designed for the urban unemployed, retired, elderly, students and children; and the new rural cooperative medical scheme (NRCMS) for rural residents [2]. After 2012, the government started to use surplus funds from UEBMI and NRCMS to purchase serious illness insurance from commercial health insurance institutions for urban and rural residents and provide subsidies to enterprises that purchase commercial health insurance for employees. While China’s social medical insurance system provides near universal coverage, after more than 20 years of exploration and experimentation patients still face high out-of-pocket medical costs, which imposes a healthcare costs burden on the sick. Against the background of more than two decades of healthcare reform and development, commercial health insurance can support and improve China’s social medical insurance system. Understanding the use of healthcare big data by China’s commercial health insurance companies is a research lacuna, especially given the difficulties currently faced by China’s healthcare system. We offer an interim assessment of healthcare big data use in China’s commercial insurance industry, which is both urgent and so-far largely neglected.

Given that healthcare financing is a local government responsibility, there are significant differences and uncertainties between different geographical regions in terms of financing arrangements, level of healthcare investments, waiting lists, insurance coverage and different quality of community, county and tertiary hospitals. With increasing population aging, where 28% of China's population will be aged 60 and over by 2040, or an estimated 402 million elderly [2], health insurance and the health system will face rapid increases in the number of chronic diseases, such as cardiovascular disease, cancer and diabetes [3]. The impact on the demand for maternal and child healthcare is uncertain due to China’s three-child policy [4]. One certainty is that China's total health expenditure will continue to grow [5]. To cope with these rapidly growing pressures on China’s social medical insurance expenditures and services, including the challenge of social fairness, one solution is to enhance the commercial medical insurance industry.

One major constraint on the development of commercial health insurance has been asymmetric information between commercial health insurance companies and the insured, giving rise to moral hazard and adverse selection risks. When prospective policyholders provide incorrect information, such as concealing pre-existing health risk information, or insurers have incomplete information on policyholders, commercial health insurance companies face challenges in product design and development, product pricing, sales cost management, insurance premium determination and risk management control. These challenges saw Chinese commercial insurers’ business growth stall and commercial health insurers’ products fail to meet policyholders’ expectations.

One way of addressing these challenges to commercial insurance is through healthcare big data, which refers to healthcare related data generated in the process of people's disease prevention and health management [6]. Big data are complex datasets that traditional data processing system cannot efficiently and economically store, manage, or process. Characterised by volume, variety, velocity, veracity, and value, big data technology supports a wide range of health care functions, including clinical decision support, population health management, and disease monitoring. By discovering correlations in data and understanding patterns and trends, big data technology can improve health care, save lives, and reduce health system costs. Through the analysis of patient characteristics and patient nursing costs, the most clinical cost-effective treatment methods can be determined; the application of big data analysis technology to patient files can identify individuals who may benefit from preventive care or lifestyle changes; the collection and analysis of medical procedure data can determine the most valuable patient nursing programs; during pandemics, big data can alert, track, and trace infected individuals; and through analysis and drug treatment data, the health status of the population can be monitored [7]. Based on healthcare big data, China's medical and health commercial insurers have promised to expand the supply of medical resources, control medical costs and improve their operational efficiency and medical services quality. Importantly, healthcare big data can reduce insurer-insured information asymmetries and improve the quality of commercial insurers’ medical services decision-making efficiency [8].

Outside China, there are studies on the healthcare big data experience of insurers [9–13], such as the U.S. Clover Health and United Health Group [14, 15], on the application of healthcare big data from a theoretical perspective [16–19] and on the development of commercial health insurance products based on big data technology [20]. Of course, different countries’ medical and health insurance systems mean the importance and operation of commercial health insurance in one country will diverged from that in other countries. Further, different degrees of informatization mean the access to healthcare big data differ across countries. These country and institutional differences mean that overseas healthcare big data experience provides only limited guidance to understanding China’s very different healthcare big data and commercial health insurance systems. Given China’s unique medical insurance system, informatization process, specific policy environment and technical constraints, this paper analyzes the application of healthcare big data in commercial health insurance companies in China. It sets out the potential methods used in healthcare big data insurance; analyzes the shortcomings of existing healthcare big data in commercial health insurance; and assesses the obstacles and risks in the implementation of healthcare big data in commercial health insurance. The paper sets out specific action plans for Chinese commercial health insurance companies to improve their management level and business performance and recommends actions for government policymakers to promote the development and financing of China’s commercial health insurance industry.

## Methods

**Fig. 1:**
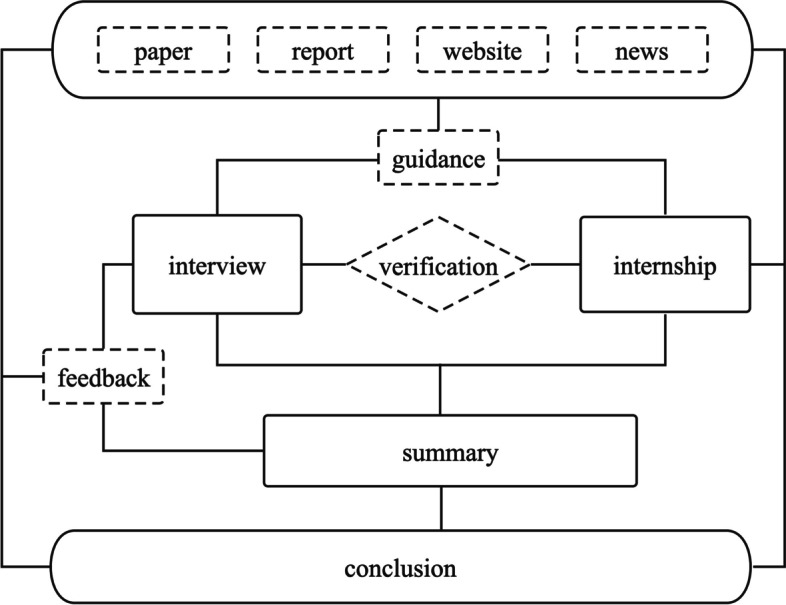
Research method

As set out in Fig. 1, this research is mainly carried out through interviews and accessing academic papers, research reports, news reports and website reports.

**Fig. 2:**
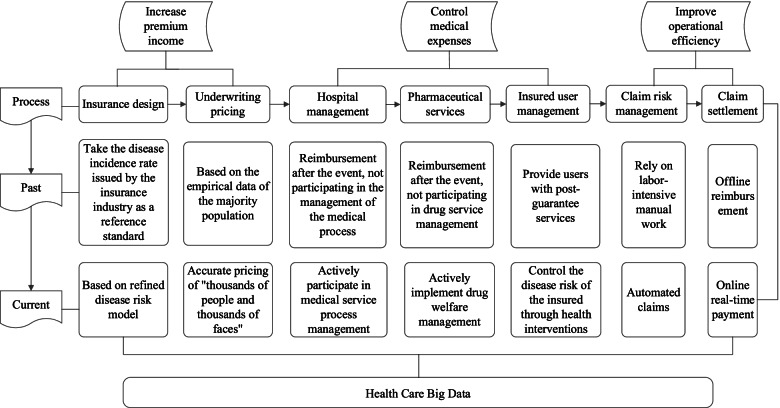
Role of healthcare big data in each link of commercial health insurance product cycle

As shown in Table 1, we undertook interviews with health management personnel, including actuaries, product managers, business executives and information technology personnel at commercial health insurance enterprises. For commercial health insurance enterprises, interviews investigated service processes; the specific responsibilities of management personnel; scope of managers work; collection and use of healthcare big data; and views on the future of healthcare big data. Table 1 also sets out data sources, including research papers, government websites, reports by the Insurance Association and insurance companies, industry and government health insurance surveys, news reports and public information disclosure documents on commercial health insurance. Based on these qualitative and quantitative data, Fig. 2 depicts the life cycle of commercial health insurance products, which we categorized into three main roles: increasing the premium income, controlling the payment of medical expenses and improving the operation efficiency of commercial health insurance. Each role in Fig. 2 is divided into different processes, comprising insurance design, underwriting pricing, communication management of medical institutions, drug service, insured user management, claim risk control and claim settlement. Figure 2 also sets out the past practices of commercial health insurance enterprises in each link of the life cycle. Finally, we assessed the current effect of healthcare big data on each link of the commercial health insurance product life cycle in Fig. 2.

**Table 1:** Sources Healthcare big data

Perspective	Contents	Data sources
Traditional	Traditional operation mode of commercial health insurance	Interview report on general manager, product director, actuary, claims director and technical director of commercial health insurance company Interview report on sales director and claims director of commercial health insurance brokerage company Interview with hospital directors and doctors Interview report on drugstore owners and doctors Interview report on senior managers of physical examination institutions Interview report on senior managers of PBM company Interview report on senior managers of commercial health insurance third-party service companies Academic papers Report issued by insurance institution Research Report of securities company Report issued by the Insurance Association
Trial and application results	Application of healthcare big data in commercial health insurance	Interview report on general manager, product director, actuary, claims director and technical director of commercial health insurance company Interview report on sales director and claims director of commercial health insurance brokerage company Interview with hospital directors and doctors Interview report on drugstore owners and doctors Interview report on senior managers of health insurance product R & D company Interview report on senior managers of physical examination institutions Interview report on senior managers of PBM company Interview report on senior managers of commercial health insurance third-party service companies Reports from Baidu and Sina News Academic papers Report issued by insurance institution Research Report of securities company Report issued by China Medical Information and big data Association Interview report with big data business analysts
Difficulties and opportunities	Problems and opportunities of healthcare big data in the application of commercial health insurance	Interview report on general manager, product director, actuary, claims director and technical director of commercial health insurance company Interview report on sales director and claims director of commercial health insurance brokerage company Interview with hospital directors and doctors Interview report on drugstore owners and doctors Interview report on senior managers of health insurance product R & D company Interview report on senior managers of physical examination institutions Interview report on senior managers of PBM company Interview report on senior managers of commercial health insurance third-party service companies Academic papers Report issued by insurance institution Research Report issued by securities company Government work report

## Results

### Increase premium income

The first role in the life cycle of commercial health insurance is premium income that compensates the insured for direct health expenses or indirect losses due to injury caused by disease or accident. Premium income must be sufficient to guarantee business viability. Based on a comprehensive survey of the development of commercial health insurance in 36 large and medium-sized cities in China, the China Insurance Industry Association’s "2018 China Commercial Health Insurance Development Index Report” found only 6.7% of the residents in China purchase commercial health insurance, and the coverage rate was less than 10% of the population [21]. Insufficient supply of commercial health insurance was the main reason for the low coverage, limited by insurance products directed to healthy people. The World Health Organization (WHO) defines health as not only the absence of disease or weakness, but also the physical, psychological state and social well-being [22]. Only 15% of the Chinese meet the definition of health by the WHO; 15% of the people are in a state of disease; and the remaining 70% are in a state of sub-health, displaying no diseases, but feeling unwell, fatigued, reduced vitality and adaptability, and experiencing confusion, boredom and helplessness [23]. The lack of effective actuarial data to support the development and pricing of commercial health insurance products meant commercial insurers experienced moral hazard and adverse selection problems, where the insured have better and different information than commercial health insurers and the insured provide misleading health information or act opportunistically once insured. Healthcare big data promises to provide the opportunity for commercial health insurers to price products to attenuate the moral hazard and adverse selection problems.

The first element in increasing premiums in Fig. 2 is product design, which depends on accurate data on the incidence rate of diseases in China. However, the statistics of China's disease incidence rates are imperfect, which provides limited basis for actuaries to design products. Healthcare big data provides the opportunity to build disease risk models and medical expense models based on disease population statistics, disease risk factors and diagnosis and treatment path analysis. Through disease risk models, commercial health insurers can better understand the influencing factors of diseases, so product design allows more targeted health management services, reduces the probability of disease occurrence in insured customers, and reduces the loss ratio of commercial health insurance products in general. In addition, disease risk models allow a better understanding of the incidence probability of disease among the insured and the impact of related disease complications, so that commercial health insurers can have a more accurate understanding of the contingent risks when designing insurance products for related special diseases. Second, the medical expense model estimates different diagnosis and treatment effects and costs for different diagnosis and treatment paths. For example, the fitness and physical condition of residents in different geographical areas differ more than that between residents living in the same area, which means the medical insurance needs more regional data. Through the analysis of healthcare big data, commercial health insurers can evaluate the differences in the occurrence of diseases in different regions and customers’ diverse demands for existing and new insurance products.

The second element in Fig. 2 under premium income is underwriting pricing. The traditional pricing method of commercial health insurance is based on experience. To ensure that it can bear the risk of future claims, commercial health insurers charge relatively high premiums to the high-quality insured and refuse to insure potential high-risk applicants. Since the potential client’s health information cannot be obtained in a timely and accurate manner, the asymmetric information problem arises when it is not possible to accurately assess whether the customer is a high-quality customer or a high-risk customer. Underwriting pricing is particularly sensitive to the moral hazard and adverse selection problem, where the true health characteristics of potential policyholders is difficult to assess a priori. Commercial health insurance companies determine the next insurance rate based on the previous compensation results and information on prospective customers. Without healthcare big data, there will be a vicious circle of increases in the insurance premium rate for high-risk customers, which reduces the insurance willingness of high-quality customers, to cover potential losses due to high-risk customers.

Based on healthcare big data analysis, commercial health insurers can better predict and assess risks through their data-driven models, determining more accurate premiums where the premiums paid by healthy people are lower and the premiums paid by people with underlying health conditions are higher. By making use of applicants’ healthcare big data, insurance companies can estimate more accurately the systematic risks of potential applicants in the underwriting process. By analyzing an applicant’s healthcare big data, especially medical records, commercial insurance companies can assess a large amount of relevant information on potential clients.

By investigating an applicant’s previous insurance and medical records held by other insurance companies or hospitals, commercial health underwriters can more effectively analyze an applicant public health treatment record, including a record of purchasing high-risk insurance products, such as repeat insurance and short-term large amount insurance. In Fig. 2, this is labelled “thousands of faces, thousands of people”, where aggregated individual data are used for underwriting pricing. For example, Zhongan online, China's leading commercial health insurance company, launched the first domestic health insurance product, bububao, which combines wearable devices and sports big data in 2015. The main feature of bububao is that it has changed the previous rough pricing mode of health insurers with age and sex as the main pricing factors, and introduced the sports factor (including wearable healthcare big data devices, such as WeChat steps) as a way of pricing commercial health insurance premiums [24]. In October 2019, Ping An Health Insurance Company launched hellorun health credit, which assesses the premium margin of the product through the insured's daily health behavior, including WeChat steps, heart rate, and weight. The higher the user's health behavior index and health status index, the higher the discount rate of health insurance products, up to 30% limit [25].

### Controlling medical expenses

**Fig. 3:**
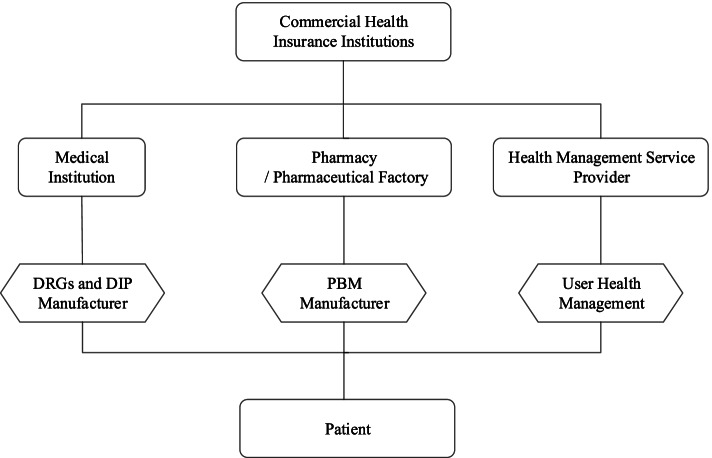
healthcare big data links with managed medical insurance system

Figure 2 identifies controlling medical expenses as the second role of healthcare big data. The traditional operation mode of commercial health insurers is only to perform the function of post-healthcare reimbursement. Commercial health insurers have limited ability to assess the medical service content provided to the insured. Over-use by patients and over-servicing by hospitals and medical providers shape the medical costs insurers compensate their clients. Managed medical insurance matches the provision of medical services to the insurer’s funds paid to hospitals performing the medical services. The core of the managed medical model is that commercial health insurers and medical service providers become a community of interest to mutually control risks and reduce costs. As shown in Fig. 3, the medical service providers include a range of medical institutions, including hospitals, community health centers, nursing homes, emergency centers and school infirmaries, pharmaceutical manufacturers, pharmacies, health management service providers, such as physical examination centers, health consulting institutions and health information service providers. In the managed medical insurance system, healthcare big data sharing requires links and good communications between commercial health insurers and medical service providers. Through healthcare big data, commercial health insurers can assess and provide feedback to change of medical service providers’ actions, controlling medical expenses without impacting appropriate diagnosis and treatment.

Based on Figs. 2 and 3, DRGs (diagnosis related groups) and DIP (diagnosis-intervention packet) are the first elements to help control medical expenses. DRGs and DIP have been adopted by medical institutions, which provides potential big data connections between commercial health insurers and medical institutions. DRGs are precise and scientific medical payment methods, comprising a patient classification system that standardizes prospective payment to hospitals and encourages cost containment initiatives. Patients are divided into different DRGs according to the patient's age, disease diagnosis, complications, treatment methods, disease severity and prognosis, and DRGs undertake direct settlement for services with medical institutions [26]. The efficiency and cost management of DRGs depends on healthcare big data. The implementation steps of DRGs payment methods include data collection and analysis, grouping, pricing and dynamic iteration, where the success of DRGs depends on a large amount of clinical data and disease cost accounting data. The more accurate and richer the data, the greater the efficacy of DRGs. The DIP score payment system depends on the statistical correlation between a disease and its treatment through medical record data, calculated by region. The basic data required by DIP includes multi-dimensional information, such as disease coding system, resource consumption, treatment mode, disease severity and medical status [27]. Commercial health insurers have a weak influence on medical institutions, mainly due to the lack of tools and data to effectively evaluate the quality of medical services. DRGs and DIP effectively evaluate the quality of medical services provided by medical institutions, which clarify the medical costs and medical treatment quality of each disease group, improve the transparency of medical service provision, and facilitate commercial health insurers to monitor hospital expenditures and identify (in)efficient services. Such big data can attenuate the widespread problem of medical treatment over-servicing, encourage and guide high-efficiency and low-cost medical service provisions, and promote the competition among hospitals.

From Fig. 3, the pharmacy benefit management (PBM) element in controlling medical expenses is a professional third-party drug management service [28]. As a third-party management organization, PBM coordinates the relationship among pharmaceutical enterprises, hospitals, pharmacies and commercial health insurers to reduce the overall drug expenses. Using PBM, the insured go to a designated hospitals for medical treatment and purchase drugs in a designated retail drugstore (or door-to-door service) with the prescription issued by a doctor. PBM supports the interests of commercial health insurance, through the development of a drug catalogue, prescription audit and other drug management systems.

The key factor of PBM success is medical informatization, dependent on healthcare big data and industry integration, helping commercial health insurers achieve real-time prescription audit and settlement reconciliation information. The core resources of PBM are the large amount of clinical data accumulated by the hospital information system, data from the PBM drug catalogue management and prescription audit system, and PBM’s retail pharmacy and pharmaceutical factory negotiated price network. For example, Mgxin health company has built yaoyibao, a comprehensive PBM platform for commercial health insurance companies. Yaoyibao has established cooperative relations with several commercial health insurers, launched dozens of drug insurance products, and provided pharmacy planning, pharmacy direct payment service, assistance medication application guidance service and outpatient green pass services [29].

The last element in controlling medical expenses in Fig. 2 is health management services, which is a comprehensive and continuous detection, evaluation and intervention system tracking an individual’s or a group’s health and risks status. Health management services include health prevention, health promotion, lifestyle management, chronic disease management, medical demand management and rehabilitation, which can prevent and delay the occurrence of diseases. Health management measures facilitate commercial health insurers transfer of financial risks, control medical costs and attenuate information asymmetry. For the insured, it can reduce the risk of illness, identify symptoms of diseases in advance and reduce expenditure on disease treatment costs. Preventive medicine research and practice mean that spending RMB1 on prevention and health management can save RMB8.59 on medical expenses and save RMB100 on non-medical costs, such as lost work time [30]. According to Harvard’s Center for Disease Prevention, 80% of heart and diabetes, 70% of stroke and 50% of cancer can be avoided through health management [31]. Compared to waiting until the physical disease occurs before treatment, active health management can detect diseases in advance, reducing the medical cost of maintaining a person’s health The data above are also applicable to an individual's management of their own health [32].

**Fig. 4:**
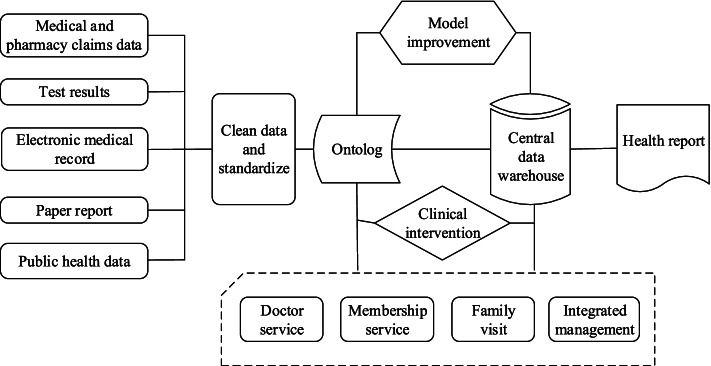
Big data analysis process in health management services

While healthcare big data provides important information to support for health management services, using healthcare big data for health management services remains a complex process. It is necessary to fully consider the data source, data storage, data standard, data cleaning and data processing models, and carry out clinical intervention on the insured based on these data. As shown in Fig. 4, the research on the influencing factors of chronic diseases is relatively mature, which means that healthcare big data analysis can help predict the complications of diseases. Identifying the risk of complications of the insured through data analysis and early health management intervention can greatly reduce medical expenses and improve the populations overall health level. Disease prediction based on healthcare big data takes the insured's electronic medical and drug prescription record, pathological test data and image data as input data, and predicts the probability of complications of the insured through machine learning methods. After identifying the high-risk population, health management service providers can regularly carry out on-site physical examination for the insured, or remind the insured to continue when a certain examination or treatment is interrupted, so as to help the patient avoid hospitalization [33].

### Healthcare big data and operational efficiency

**Fig. 5:**
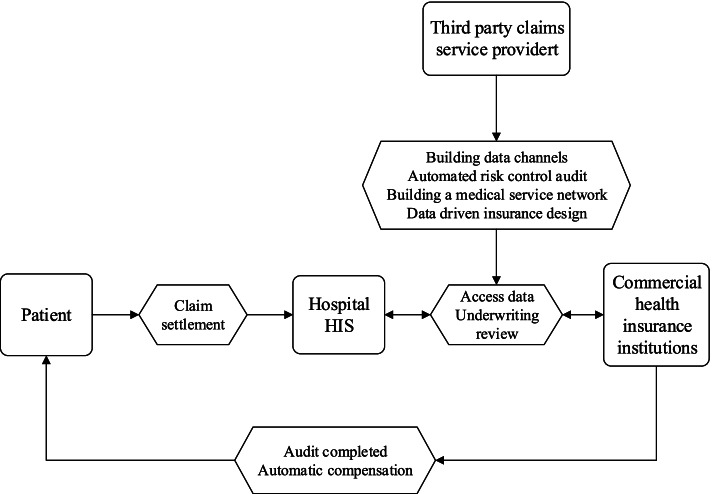
Role of healthcare big data in direct payment of settlement of claims

The third major role of healthcare big data in Fig. 1 is operational efficiency, which has two elements, claim risk management and claim settlement. The core of the insurance business lies in the high frequency and large number of claims. The traditional claim settlement process for commercial health insurers occurs after policyholders first apply for compensation from URBMI or UEBMI, and then apply to commercial health insurance companies using the relevant hospital documents. The claimants face challenges in getting their claims settled, experience slow reimbursements and cumbersome claim procedures and frequently suffer a poor customer experience. For commercial health insurers, offline claims verification has problems, such as scattered users, high difficulty in claims verification, slow claim verification speed, and high administration compensation cost. The traditional claim cycle method can take more than 40 days, with claim settlement personnel frequently sorting through more than 7 different records and more than 10 documents [34]. An investigator for the commercial health insurer can visit up to three different hospitals a day, with the cost of a single medical record retrieval as much as RMB300, and the time for completing the retrieval of medical records taking 5 days or longer [34]. Using healthcare big data, direct claim payment means that when the insured leaves the hospital there is an automatic deduction and reimbursement of insurance expenses. The essence of claim direct payment is to transform the health insurance claim service from "offline labor-intensive" to "online automation", which can significantly reduce the operating costs of commercial health insurers and improve customer experience. Big data allows commercial insurers to directly access the client’s digital information using the hospital information data system, which significantly reduces the cost of traditional offline recording and claims procedures, but also manages potential fraud. Figure 5 diagrammatically depicts the automatic claims process, which uses healthcare big data to save reimbursement costs, to shorten the claim cycle and to reduce the probability of insurance fraud caused by tampering with the paper documents.

## Discussion

### Evaluations

#### Health care big data with unified standards, privacy protection and controllable risk

The most efficient way for commercial health insurers to obtain health and medical data is through cooperation directly with medical institutions, which has the advantages of high data quality, strong real-time properties and scenario planning. The challenge lies in the low rate of cooperation between medical institutions, especially public hospitals, and commercial health insurance companies. The low rate of cooperation means high communication cost and low scale effects. The second challenge to accessing medical data is cooperation on market-oriented data platforms, which has the advantages of higher docking efficiency and lower cost, is poor data granularity; issues of data quality and effectiveness; and the hidden dangers in the stability of cooperation due to the influence of compliance factors. The third challenge to accessing medical data is the poor cooperation with pharmaceutical enterprises, equipment manufacturers and technology companies that limits access to medical data, individual and group health and behavioral data and medical research data, all which supports commercial insurers’ product pricing and health management. The advantages of this cooperation lie in the diversity of data dimensions and low sharing cost, but barriers exist related to the problems of real-time authorization and data dispersion.

At the national level, although there are regional data centers, problems of data management and ownership mean that effective mechanisms for health data sharing with commercial health insurers have been limited. There are many market participants in the construction of healthcare big data, but the resources of the various channels are scattered, which makes it difficult to form scale effects. The adverse effects of poor data sharing are inefficiencies in cost savings and product and service innovation.

#### Data security: data sharing and data mining pose privacy risks

Since healthcare big data involve personal genetic information, disease history, drug use history, family history and other sensitive information, privacy security poses a major challenge to health data sharing. The more complete the shared data, the greater the potential value, but the greater the risk of privacy leakage. For example, healthcare big data can be applied to the research and development and sales and compensation processes only after data mining. Through data mining technology, private information may be exposed and leaked to third parties.

#### Data standard differences

The diversity of healthcare big data means variations in data quality and reliability. Data from different information systems in different hospitals, with nonstandard clinical case records, result in diverse data standards, which makes healthcare big data difficult to analyze. Ninety percent of healthcare big data comes from medical institutions, while the information system of medical institutions developed earlier and the standard was formed later, resulting in complex data formats of medical institutions. Taking the hospital and medical information systems, there are more than 2000 medical information providers and hospitals in China with different data formats, which inhibits data fusion and sharing [35].

#### Data quality needs to be improved

Data quality dimensions include integrity, consistency, rationality, timeliness, uniqueness, effectiveness and accuracy. Big data quality is the premise of big data research and the basis of all data analysis, data mining and data decision-making. In the case of problems in data quality, it is difficult for advanced data mining analysis models and tools to find the valuable information contained in the data. Especially in the field of healthcare big data, the integrity, accuracy and timeliness of healthcare big data are difficult to manage and control due to different data standards in different regions and different medical institutions. Data quality management is a complex system engineering challenge, including the whole life cycle process of data generation, collection, conversion, storage, transmission and use. The efforts of the entire related medical system and medical industries are required to improve data quality from the aspects of policy, personnel and technology.

### Recommendations

#### Healthcare big data with unified standards, privacy protection and controllable risk

Successful healthcare big data sharing needs not only the guarantee of the technical means of data transfer, but also a framework of regulations and policies. At present, several regional healthcare big data platforms in China are actively developing policies for big data sharing. These platforms aim to share healthcare big data, provide an important data basis for regional disease risk intervention (such as chronic diseases) and epidemiological research (such as influenza and Covid-19), and improve patient health. We recommend that national, regional and local government departments coordinate to introduce policies to facilitate the cooperation between commercial health insurance companies and regional healthcare big data platforms. Those underdeveloped regions with lagging technology should be provided extra government support to promote the development of healthcare big data, explore the construction of national healthcare big data platforms, and promote the cross-industry application of healthcare big data [36]. In terms of technology, we recommend the establishment of data sharing platforms and data exchange mechanism across institutions and regions according to nation-wide standards and specifications. For example, data providers and data users can sign a data sharing agreement (DSA) to clarify the main responsibility, working mechanism, sharing time, sharing scope, sharing mode, sharing process and benefit distribution in advance and promote the establishment of a publishing and citation system for shared data. DSAs will require government support and oversight. The aim would to be compile resource catalogues and analyze business requirements; integrate resource catalogues across regions through a standard exchange system; and form a distributed catalogue management system that is physically dispersed and logically centralized.

#### Standard specification system

As noted above, government management departments should establish a healthcare big data standard and specification system, promote the construction of healthcare big data and ensure the integrity, authenticity and reliability of data. This requires government cooperation across national, regional, provincial and local levels, which marks a major change in China’s provincial and local system of healthcare. Government should play a leading role in the establish of a data standard system for medical and health institutions, physical examination institutions and third-party diagnosis and treatment institutions; improve the coding of various disease classification, unify medical terms and formulate inspection and diagnosis standards; standardize the collection, classification, application and sharing of big data; and accelerate the standardization and integration of the establishment of healthcare big data. The healthcare big data standard system should consist of five parts: basic standards, technical standards, product and platform standards, data security standards, application and service standards. The basic standards provide the whole standard system with general principles, terms and reference models and metadata. Technical standards regulate healthcare big data related technologies, especially data governance and data quality. Data governance standards mainly aim at data collection, pre-processing, analysis, visualization, access and capability maturity evaluation modelling. Data quality standards puts forward specific management requirements and corresponding index requirements for data quality to ensure the quality of data in the process of generation, storage and evaluation, and secondly, in the exchange and use of various aspects of the quality, including quality evaluation, data traceability, quality testing and other standards. Product and platform standards regulate the technology products and application platforms related to healthcare big data, including relational database products, unstructured data management products, intelligent tools, visualization tools, data processing platforms and test specifications. Data security standards mainly include general requirements and privacy protection. Application and service standards regulate the applications and services provided by big data from the aspects of technology, function, development, maintenance and management, mainly including open data sets, data service platforms and related data sets. The open data set standard mainly regulates the content and format of the open data package provided to a third party. Data service platform standards are a functional, maintenance and management standard for big data service platform. Related data sets refer to the special data standards generated according to the characteristics of the medical and health field [37].

#### Data quality management system

Data governance is a collection of activities that exercise power and control over data asset management. We recommend data quality continuous improvement management mechanisms that combine technology and management. Data governance runs through the whole process of data management, such as data architecture management, data development management, metadata management, master data management, document and content management, data operation management, data quality management, data security management, guiding the implementation of data management functions [38]. Data governance is the most onerous task of building a healthcare big data sharing platform. Building a healthcare big data governance system is the basis of data governance. There is a key role for government in the establishment of a healthcare big data governance systems, which should include strategic objectives and planning, organizational structure, policies and systems, process specifications, data standards, technical platforms and tools, supervision and assessment.

#### Data security and privacy protection

In the application of big data, the protection of personal information and privacy should be a central concern for the health industry. We recommend that the China Banking and Insurance Regulatory Commission and the Insurance Association of China should provide regulation and oversight of the whole process of the collection, storage, processing and application of personal health and medical data by commercial health insurance institutions. All commercial health insurers should have regulations for "customer authorization", "privacy data protection", "data security", "data sharing inside and outside the industry", "data application scope", "application subject" and "process and method" [39]. Government regulation should oversee the commercial health insurance institutions’ establishment of a data security management system to monitor and supervise the privacy of personal data; establish integrity files or credit evaluation records, pay attention to content security and technical security.

These recommendations must recognize the challenges of integration, management and analysis faced by big data. We promote the advantages of logical data warehousing, a method to realize data virtualization. In terms of big data management, we identify event flow processing and real-time data analysis technology to distinguish business-related information from irrelevant information, reduce storage and processing costs, and attenuate the burden of data integration framework. In terms of big data analysis, memory and database computing do not need to put various data sources into the standard data model before analyzing data, which can speed up data analysis. In big data security, one way to innovate data utilization is to use blockchain technology to build a neutral third-party security housing platform to realize the separation of ownership and use-rights. Secure house platforms allow the demand side to use data that it does not own. For the data supplier, secure house platforms provide massive data access to multiple data sources. For the data demander, secure house platforms use data fusion technology to provide multi-party high-quality data resources that the data demander did not have previously [40]. It also supports the third-party algorithm and user-defined algorithms.

There are a number of limitations and future directions to our study. Given that Chinese commercial health insurance companies are still in the process of exploring the use of health big data, and have not yet formed a complete healthcare big data dataset, it is impossible to quantitatively measure the contribution of healthcare big data to commercial health insurance institutions. Quantification of healthcare big data needs to be addressed in future research. Due to Covid-19 epidemic, the number of on-site commercial health insurance company visits were limited. Future research should increase the number of interviewed technicians and the investigation cycle for commercial health insurance companies should be extended. Our interviews revealed limited knowledge about healthcare big data, and future researchers should assess the technical expertise of commercial health insurance technicians. Studies of specific commercial health insurance companies should be undertaken to compare and contrast the behavior and experience of individual companies. Finally, our study revealed the need to assess how new computer technology impact the application of the big data process.

## Conclusion

Healthcare big data can play an important role in the development of China's commercial health insurance industry. Healthcare big data can increase commercial health insurers’ financial viability while providing improved, and cost-effective, products and services. By providing more and better information to insurers, healthcare big data addresses the asymmetric information problem, addressing moral hazard and adverse selection problems. By combining hospital and medical organization management information systems with insurers’ data management, healthcare big data can help insurers set sustainable premiums, control medical costs and promote operational efficiency. At present, the informatization degree of China's healthcare industry remains limited. To improve the performances, products and services of commercial health insurers, we recommend government reforms in healthcare big data, such as expanding medical industry cooperation; further developing the processes of applying healthcare big data; augmenting data sharing; addressing privacy risks; setting data standards; and improving data quality.

## Data Availability

The datasets used and/or analysed during the current study are not publicly available because consent was not obtained from participants that would allow for public storage of the data. The datasets used and/or analysed during the current study are available from the corresponding author on reasonable request.
